# Outcomes of a novel double-stapled anastomotic technique in esophagectomy

**DOI:** 10.1016/j.xjtc.2024.08.007

**Published:** 2024-08-29

**Authors:** Caroline M. Godfrey, Eric L. Grogan, Hannah N. Marmor, Sigrid L. Johannesen Ringenberg, Caitlin Demarest, Eric S. Lambright, Jonathan C. Nesbitt

**Affiliations:** a Department of General Surgery, Vanderbilt University Medical Center, Nashville, Tenn; b Division of Thoracic Surgery, Tennessee Valley Healthcare System, Nashville, Tenn; c Department of Thoracic Surgery, Vanderbilt University Medical Center, Nashville, Tenn; d Department of Thoracic Surgery, Baylor University Medical Center, Houston, Tex

## Abstract

The double-stapled (*DS*) anastomotic technique associates with lower odds of anastomotic leak and stricture. *SS*, Single posterior stapled.

**Figure:**
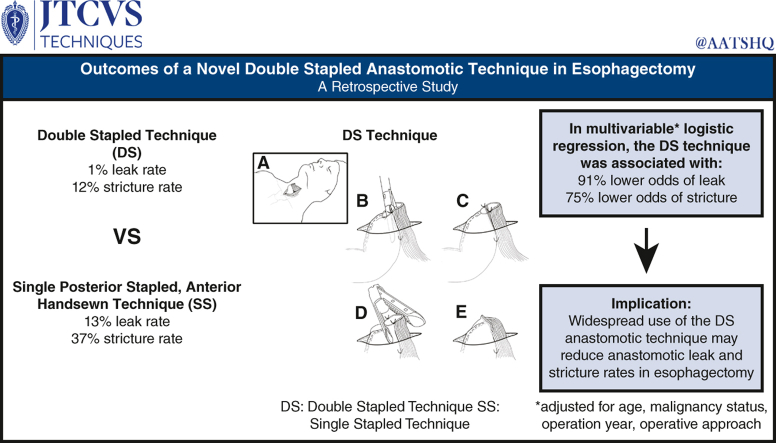

**Figure:**
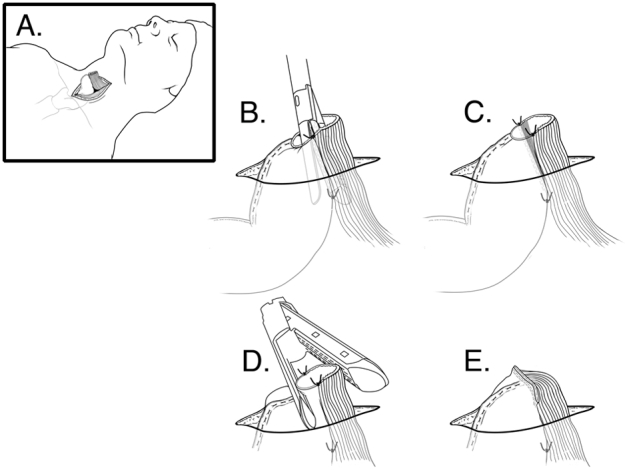
The double-stapled anastomotic technique creates a wide *V*-shaped anastomosis.

**Central Message**

The double-stapled esophagogastric anastomotic technique demonstrates lower anastomotic leak and stricture rates compared with the traditionally performed partially hand-sewn technique.

Esophagectomy is central to definitive treatment for localized esophageal cancer. Although a critical element of curative therapy, esophagectomy has significant potential morbidity and mortality.^E1^ Esophagogastric anastomotic leak (AL) rates range from 10% to 16% and associate with higher morbidity and mortality.^1^^,^^E2^^,^^E3^ Anastomotic stricture (AS) rates range from 14% to 48% and are higher in patients who experienced AL.^1^^,^^2^^,^^E3^^,^^E4^

Our institution primarily employs 2 anastomotic techniques: a novel double-stapled (DS) technique, similar to previously described linear stapled techniques^3^^,^^E4^^,^^E5^ but with instrumental modifications to reduce AL and AS, or a single-stapled, anterior hand-sewn (SS) technique. We compare AL and AS rates between these techniques (Figure 1).

**Figure 1:**
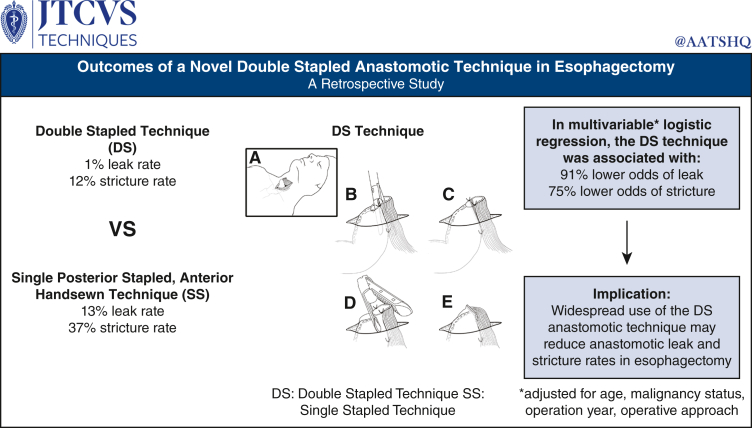
The double-stapled (*DS*) anastomotic technique associates with lower odds of anastomotic leak and stricture. *SS*, Single posterior stapled.

## Methods

We retrospectively reviewed all esophagectomies at Vanderbilt University Medical Center from July 24, 2014, to May 26, 2021. The institutional review board approved the study (#211047; approved September 23, 2021) with waiver of individual informed consent due to minimal risk. Patients who were not reconstructed, in whom the esophagectomy was aborted, or who had an anastomosis performed utilizing a different technique were excluded. Primary outcomes were anastomotic leak (defined by esophagram, computed tomography, or suspicious drain output treated clinically as leak) and anastomotic stricture (defined as anastomotic narrowing on endoscopy requiring dilation). Secondary outcomes included chyle leak, pneumonia, delayed conduit emptying (defined radiographically on esophagram), 30-day readmission, and 90-day mortality.

We compared continuous and categorical variables using 2-sample *t* tests and χ^2^ tests, respectively. We performed multivariable logistic regression comparing odds of leak and stricture between techniques, adjusting for age, malignancy status, operation year, and operative approach. Statistical analysis was performed using STATA version 17.0 (Stata Corp).

### Anastomotic Techniques

#### SS

The esophagus is transected near the thoracic inlet. Simple 3-0 Vicryl sutures are placed through the posterior wall of the esophagus and just cephalad to the planned location of the gastrotomy. A gastrotomy is made sharply, at least 1.5 cm from the original staple line created to form the gastric conduit. The posterior wall of the anastomosis is initiated with interrupted full thickness 4-0 polydioxanone to ensure approximation of the gastric and esophageal mucosa. The posterior anastomosis is then completed using a 45-mm linear stapler, utilizing 35 mm of the staple load.

The anterior opening is closed in a hand-sewn fashion with an initial full-thickness layer of 4-0 polydioxanone interrupted sutures followed by a layer of 3-0 Vicryl sutures for approximation of the gastric serosa to the esophageal remnant.

#### DS

**Figure 2:**
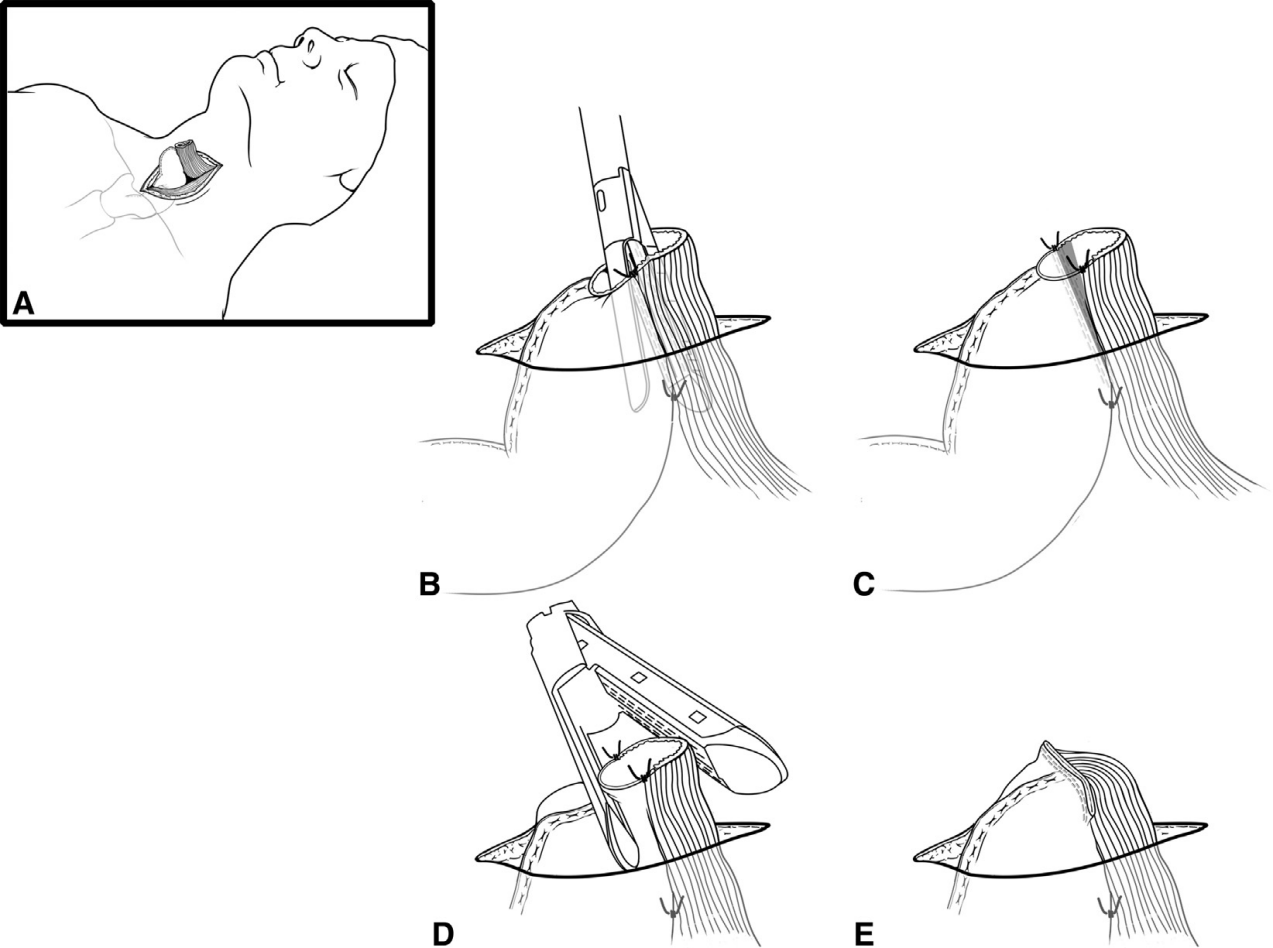
Double stapled (*DS*) anastomotic technique. A, The tip of the gastric conduit through the cervical incision, approximating the esophageal remnant. B, The linear stapler creates a long posterior anastomosis. C, A wide *V*-shaped anastomosis is formed after the first application of the linear stapler. D, the anterior opening is closed with a linear stapler positioned transversely to the posterior staple line. E, the completed DS anastomosis.

The cervical esophagus is transected a few centimeters below the thoracic inlet, leaving 10 to 12 cm remnant cervical esophagus to create a tension-free anastomosis (Figure 2, *A*). The esophagus and conduit are rotated slightly to align to the greater curvature with the left posterolateral aspect of the esophagus, allowing the staple line to be accomplished along the greater curvature, the most vascularized portion of the conduit, creating a more balanced blood supply and avoiding creation of a watershed area that could risk anastomotic integrity.

Four stay sutures are placed approximating the conduit and esophagus: 2 approximating the ends and 2 at the planned anastomotic depth to reduce tension on the staple line apex (Figure 2, *B* and *C*). The posterior staple line is created using the entire length of a 60-mm green load linear stapler placed into the esophagus and a small gastrotomy in the tip of the conduit, positioned on the mesenteric side (Figure 2, *B*). The anterior opening is closed with a 60-mm green load linear stapler, transverse to the posterior staple line, creating a wide *V*-shaped anastomosis (Figure 2, *D* and *E*).

## Results

A total of 234 patients underwent esophagectomy during the study period. Two hundred ten utilized a DS (n = 86) or SS (n = 124) anastomotic technique, comprising the study group. Five experienced thoracic surgeons performed the operations, with 3 utilizing the SS technique, 1 the DS technique, and 1 utilizing both. All surgeons employed a similar technique other than the anastomotic reconstruction. Cohort characteristics are reported in Table 1.

**Table 1:** Demographic characteristics and outcomes of double-stapled (DS) and single-posterior stapled (SS) groups

Demographic characteristic or outcome	DS (n = 86)	SS (n = 124)	*P* value
Characteristic			
Male sex	76 (88)	95 (77)	
Mean age (y)	61.8 (11.2)	61.9 (11.0)	
Race			
White	81	119	
Black	2	5	
Other/unknown	3	0	
Mean BMI	28.2 ± 8.8	27.9 ± 5.5	
Hypertension	40 (47)	79 (64)	
Hyperlipidemia	38 (44)	38 (31)	
Diabetes mellitus	24 (28)	31 (25)	
CAD	13 (15)	22 (18)	
GERD	64 (74)	76 (61)	
Barrett's esophagus	17 (20)	33 (27)	
Smoking status			
Former	45 (53)	53 (43)	
Current	20 (24)	32 (26)	
Never	20 (24)	39 (32)	
Cancer	83 (97)	107 (86)	
Preoperative chemoradiation	70 (85)	79 (74)	
Surgical approach			
Transhiatal	65 (76)	85 (69)	
Transthoracic, Ivor Lewis	9 (11)	18 (15)	
3 field, McKeown	12 (14)	21 (17)	
Outcomes			
Anastomotic leak			
Any approach	1 (1)	16 (13)	.002
Cervical anastomosis	0 (0)	11 (10)	.004
Transthoracic anastomosis	1 (11)	5 (28)	.33
Anastomotic stricture			
Any approach	10 (12)	46 (37)	<.001
Cervical anastomosis	8 (10)	41 (39)	<.001
Transthoracic anastomosis	2 (22)	5 (28)	.76
Delayed conduit emptying	9 (11)	20 (17)	.22
30-d readmission	8 (9)	13 (11)	.76
90-d mortality	2 (2)	6 (5)	.35

Values are presented as n (%) or mean ± SD. *BMI*, Body mass index; *CAD*, coronary artery disease; *GERD*, gastroesophageal reflux disease.

The AL rate was 1% (1 out of 86) for DS and 13% for SS (16 out of 124) anastomoses (*P* < .01). Among cervical anastomoses, AL rate was 0% (0 out of 77) for DS and 10% for SS (11 out of 106) techniques (*P* < .01) (Table 1). The AS rate was lower for DS (10 out of 86 [12%]) versus SS (46 out of 124 [37%]; *P* < .01). Adjusting for age, malignancy status, operation year, and operative approach (transhiatal, transthoracic, and 3-hole), the DS technique demonstrated 91% lower odds of AL (odds ratio, 0.086; *P* = .02) and 75% lower odds of AS (odds ratio, 0.25; *P* < .001). There was no significant difference in secondary outcomes measured (Table 1).

## Discussion

Esophagectomy is a frequently performed operation and cornerstone of curative therapy for localized esophageal malignancy; however, variation exists in operative approach and esophagogastric reconstruction. This reconstruction is critical because many operative complications hinge on anastomotic integrity and leaks can result in prolonged hospital stay and associated morbidity.^E3^ Literature suggests stapled techniques may improve outcomes; however, anastomotic complication rates remain unacceptably high.^4^^,^^E6^

This study describes a novel esophagogastric anastomotic technique with lower AL and AS rates than the traditional SS technique or historical reports. Similar fully stapled techniques have been described; however, there are key differences from most fully linear stapled techniques described that we believe contribute to its success and ease.^3^^,^^E5^ Most notably, the posterior anastomosis is performed on the greater curvature of the conduit, the most amply vascularized portion of the conduit. Additionally, this technique uses the entire length of the linear stapler and only a single anterior stapler application to form the anastomosis and does not require the resection of any portion of the conduit. Additionally, many of the prior studies focus on thoracic anastomoses and ours primarily focuses on cervical anastomoses, traditionally plagued with higher leak and stricture rates.E7, E8, E9

Although the DS technique has rarely been described in the literature, our result is supported by recently published literature demonstrating improved stricture rates with the DS technique compared with fully stapled anastomoses utilizing a circular stapler.^5^ This study did not include a partially hand-sewn comparison group, a technique traditionally employed in cervical anastomoses.^2^^,^^E10^ Although limited by the nonrandomized, retrospective design, our result remains significant in multivariable adjusted analyses, suggesting the DS technique could reduce esophagectomy morbidity and mortality through improved anastomotic outcomes.

## Conflict of Interest Statement

Dr Nesbitt discloses an unpaid position as the chief medical officer and on the board of Biomed Simulation that does not relate to the content of this article. The views expressed in this article are those of the authors and do not necessarily represent the views of the US Department of Veterans Affairs. All other authors reported no conflicts of interest.

The *Journal* policy requires editors and reviewers to disclose conflicts of interest and to decline handling or reviewing manuscripts for which they may have a conflict of interest. The editors and reviewers of this article have no conflicts of interest.
